# Green Space Cooling Effect and Contribution to Mitigate Heat Island Effect of Surrounding Communities in Beijing Metropolitan Area

**DOI:** 10.3389/fpubh.2022.870403

**Published:** 2022-05-02

**Authors:** Wei Liu, Haiyue Zhao, Shibo Sun, Xiyan Xu, Tingting Huang, Jianning Zhu

**Affiliations:** ^1^School of Landscape Architecture, Beijing Forestry University, Beijing, China; ^2^Department of Horticulture, Beijing Vocational College of Agriculture, Beijing, China; ^3^School of Chemistry and Chemical Engineering, Beijing Institute of Technology, Beijing, China

**Keywords:** green space, cooling effect, cooling contribution, metropolitan area, urban thermal environment, resident health

## Abstract

With the rapid process of urbanization and global warming, many metropolises are vulnerable to high temperatures in summer, threatening the health of residents. However, green spaces can generate a cooling effect to mitigate the urban heat island effect in big cities. They can also help to improve the living quality and wellbeing of surrounding residents. In this paper, we utilized the radiative transfer equation algorithm, k-means clustering algorithm, big data crawling, and spatial analysis to quantify and map the spatial distribution, cooling capacity, and cooling contribution for surrounding communities of 1,157 green spaces within Beijing Fifth Ring Road, a typical metropolitan area. The findings showed that (1) the area proportion of the heat island in the study area is larger than that of the cooling island. Accounting for only about 30% area in the study area, the green spaces reduce the average land surface temperature by 1.32°C. (2) The spatial features of green space, such as area and shape complexity, have a significant influence on its cooling effect. (3) Four clusters of green spaces with specific spatial features and cooling capacity were identified. And there were differences among these clusters in green space cooling contribution for the surrounding communities. (4) The differences in green space cooling contribution also existed in different urban zones. Specifically, the middle zone performed significantly better than the inner and outer zones. (5) We furthered in finding that some green spaces with medium and high cooling contributions need to improve their cooling capacity soon, and some green spaces with low cooling contributions or no contributions have a good potential for constructing new communities in the future. Our study could help planners and government understand the current cooling condition of green spaces, to improve their cooling capacity, mitigate the urban heat island effect, and create a comfortable and healthy thermal environment in summer.

## Introduction

With the rapid development of urbanization and global warming, urban expansion in the past led to a dramatic change in the underlying surface (1, 2). The problems of the urban thermal environment, such as the urban heat island (UHI) effect and extreme weather events, arose worldwide (3). Additionally, in recent years, the development pattern of smart growth made some metropolitan areas of high density and intensive, where the urban and environmental problems caused by high temperature also became more serious (4). According to some studies, high temperature leads to an increase in some health risks, diseases, and even mortality of the public (5–7). It also brings a vicious circle of ‘high temperature—energy consumption—“greenhouse effect”—high temperature,' which makes the urban environmental health and the ecological condition deteriorate rapidly (8). However, as an essential part of green infrastructure (GI), the urban green spaces have many ecosystem services. They can play a significant role in mitigating the UHI and promoting thermal environment health, especially in metropolitan areas, which has been confirmed by many studies (9, 10). Therefore, in the context of climate change and urbanization, how to utilize the green spaces' cooling capacity to reduce the influence of extreme heat weather in summer and improve the thermal comfort of the environment has become a hot issue for relevant scholars.

So far, the research on the green space cooling effect roughly has contained 2 aspects: the macro and the micro. The macro studies primarily focused on the spatial-temporal distribution of cooling islands (11, 12), landscape or green space spatial pattern (13, 14), and influencing factors or drivers of the green space cooling effect (15, 16). Li and Zhou found that the green space patch intensity and mean patch shape are negatively correlated with UHI intensity, while green space edge density is positively correlated with it in Illinois-Indiana-Ohio, United States (17). Zhao et al. mapped the land surface temperature (LST) and land cover of Shenyang, China, and indicated that among all types of land cover, the highest mean LST is buildings and roads, while the lowest is water. The temperature of farmland is higher than that of green space. They also emphasized that although the water and the green space have a good cooling effect, the capacity is not obvious if their area is small (3). And some researchers are focusing on the cooling effect of a certain land cover or element, such as water (18), forests (19, 20), and wetlands (21–23). Wang et al. found that LST is affected by water, and the cooling effect of water varies according to different land covers (24). In the micro aspect, most of the previous studies focused on the influencing factors of the park cooling effect in a city or the simulation of green space cooling effect in a small area (25, 26). Jaganmohan et al. measured mobile air temperature in 62 parks and forests of Leipzig, Germany, and found that the cooling quality of forests is better than that of parks. And with size increasing and shape complex decreasing, the cooling effect of parks and forests increases (27). Chen et al. defined the different aspects of parks' cooling capacities, such as cooling efficiency, cooling gradient, cooling area, and cooling intensity, and found the inequity in accessing park cooling service in Wuhan, China (28).

However, it can be seen from the previous studies that most macro studies focused on the scale of the entire city or built-up area, while the micro studies focused on the individual or group scale of green spaces. No transition appeared between the macro studies and micro studies. In addition, some macro studies ignored regional disparities in the green space cooling effect between the urban core and the suburbs, which is caused by the difference between the urban and rural structure of the underlying surface and land cover. Thus, there is a demand for mesoscale research on the cooling effect on a specific geographical area of the city. Besides, some micro research about parks' cooling effect selected fewer samples (dozens of parks), which might make the results of these studies quite inconsistent with each other in the same study area (29). And there are many green spaces in the city that are not parks, which still have a perfect cooling service for the surrounding residents. They should be included in the study samples to increase the quantity of analysis and improve the accuracy of the results. Finally, a few studies paid attention to the green space cooling contribution to the community (GCC), which partly reflects the well-being and value of green space ecosystem services. And it also can help the government and planners in a deep understanding and better management of our city.

In this paper, we selected a typical metropolitan area on mesoscale, within the 5th Ring Road of Beijing as the study area. This region with the most diverse land use, functions, and vitality in the whole city is more vulnerable to extremely high-temperature disasters. So, in this specific area, the green space cooling effect was focused on, which has great scientific value and practical significance. Then, in this paper, we quantified the cooling effect indicators and landscape indicators of 1,157 green spaces with more than 1 hm^2^ in the study area, classified them into 4 clusters with different cooling characteristics, and defined and mapped the green space cooling contribution to the surrounding communities for the first time. Our study aims to: (1) quantify and obtain the overall characteristics and spatial distribution of LST and cooling-heat islands in the study area; (2) quantify the green space cooling effect and find the spatial features of the green spaces with higher cooling capacity; (3) based on the green space spatial features and characteristics of green space cooling effect, identify different clusters of green spaces, and compare their cooling capacity and advantages; (4) quantify the spatial distribution of green space cooling contribution to communities' green spaces and assess their performance in different clusters and zones.

## Study Area

Beijing (115.7°-117.4°E, 39.4°-41.6°N) is the capital of China, located in the north of North China Plain, adjacent to Hebei Province and Tianjin city. It covers an area of 16,410 km^2^, with a population of 218.9 million in 2020 (30). And it is dominated by a warm temperate semi-humid and semi-arid monsoon climate, with hot and rainy in summer and cold and dry in winter. The annual average temperature of the city is 11–13°C, and the annual extreme high temperature is around 35–40°C.

**Figure 1:**
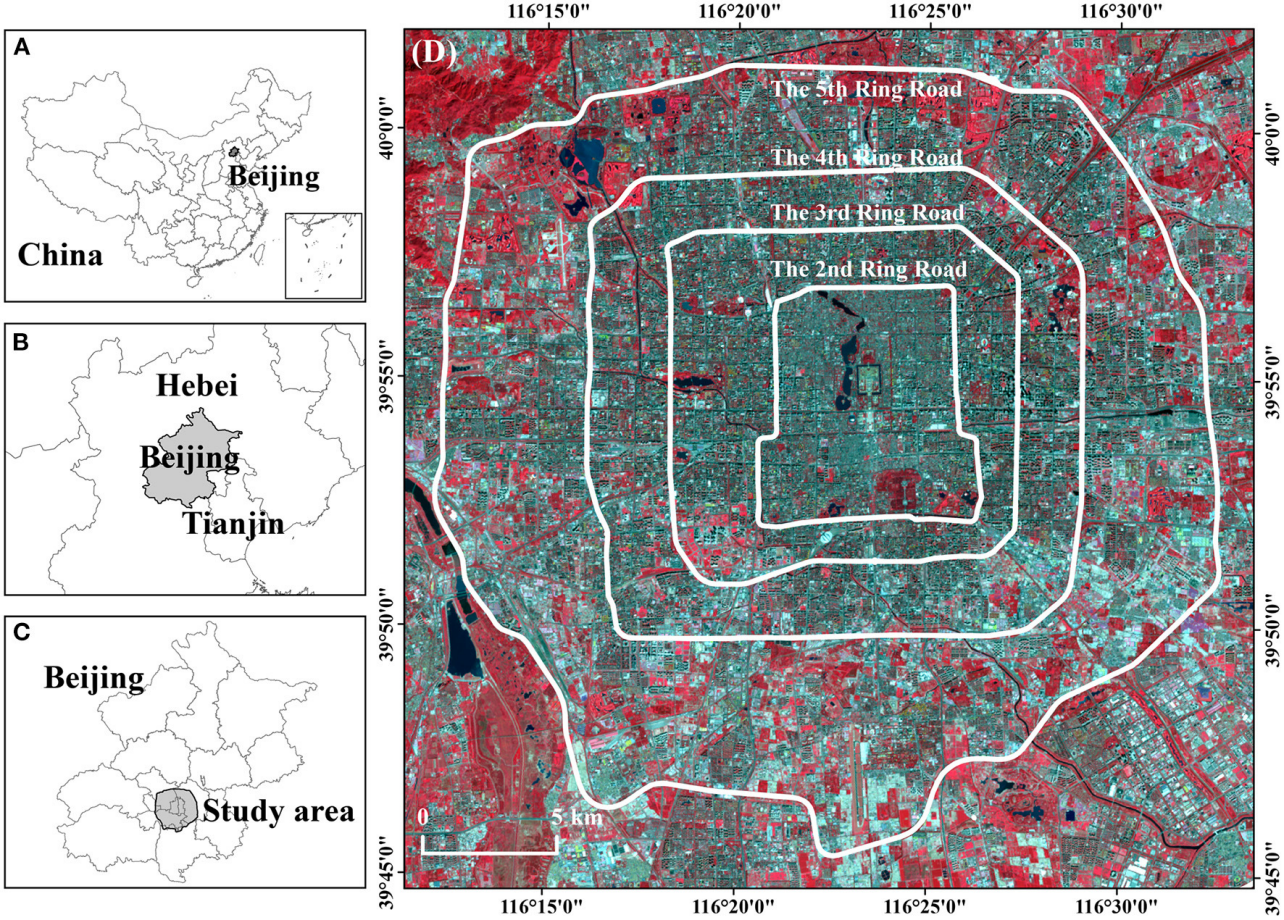
Location of the study area in Beijing metropolitan area, China **(A–C)**. The Landsat8 remote sensing image **(D)** is displayed in false color mode, which is composed of band 5 (near-infrared), band 4 (red), and band 3 (green).

The study area is within the Fifth Ring Expressway in Beijing, including the whole or part of the area of the six administrative districts, which are Dongcheng, Xicheng, Haidian, Chaoyang, Shijingshan, and Fengtai (Figure 1). This area undertakes the four core functions of the capital according to the *Beijing Master Plan (2016–2035)*, such as the political center, cultural center, science and technology center, and also international exchange center (31). In addition, our study area is densely populated and is also the core of the highly-urbanized religions of Beijing. The dwelling population in this area accounts for 48.9% of the whole city, while it covers a total area of 66,655 hm^2^, accounting for only 4.1% of Beijing. Moreover, according to the “figure-ground relation” of the city and urban development history, we divide the study area into three zones: the inner zone within the 2nd Ring Road, the middle zone from the 2nd Ring Road to the 4th Ring Road, and the outer zone from the 4th Ring Road to the 5th Ring Road.

## Materials and Methods

### Retrieving Land Surface Temperature and Classification of Cooling-Heat Islands

**Figure 2:**
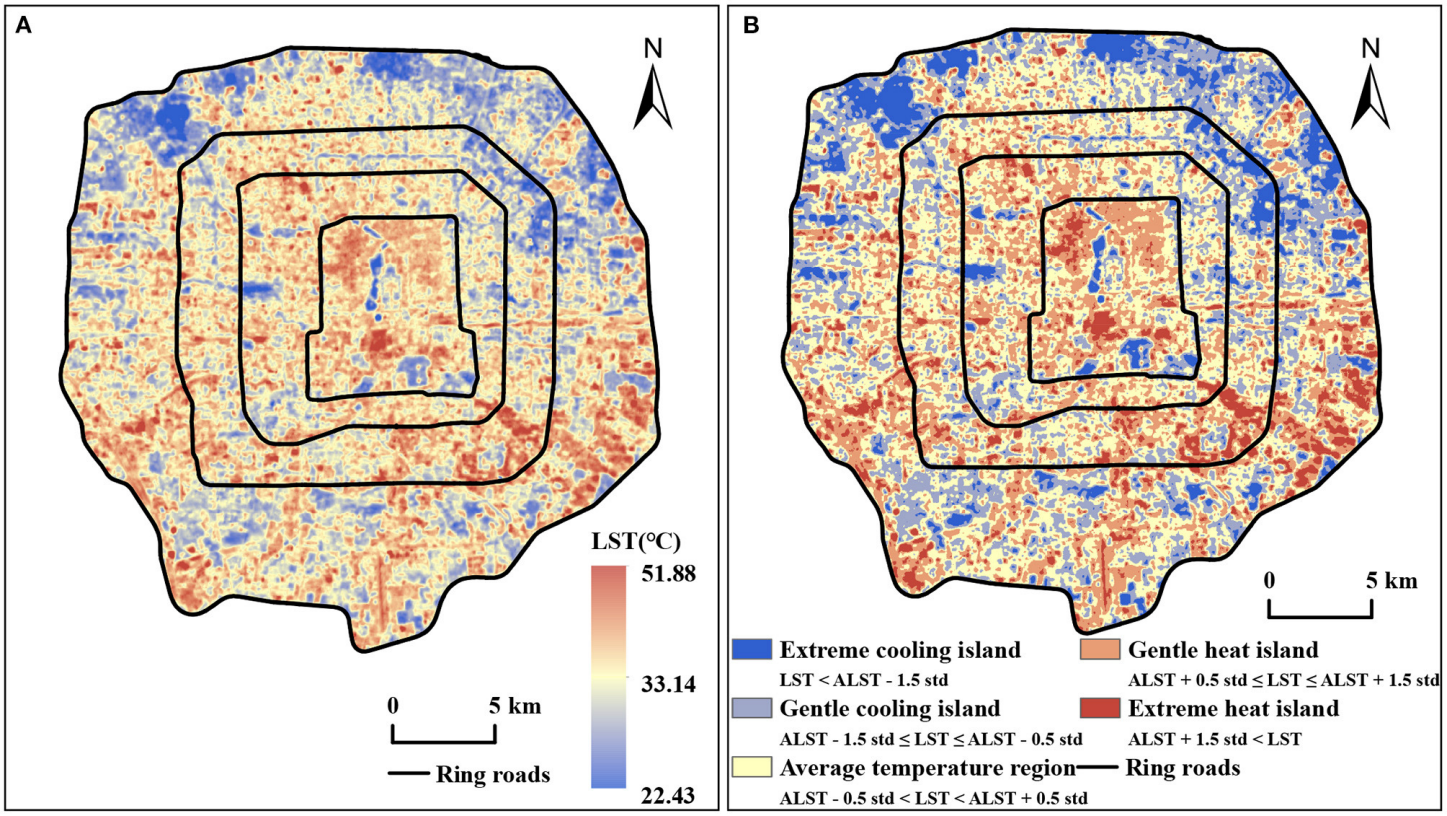
Spatial distribution of LST **(A)** and cold-hot islands at different levels **(B)** in the study area. Where ALST is the average LST; std is the standard deviation of LST in the study area.

The Landsat 8 TIRS image (path/row: 123/32, cloud cover: 1%, and no cloud in the study area) was obtained at 02:53:37 GMT on 12 September 2017. We downloaded the remote sensing data from the Geospatial Data Cloud (www.gscloud.cn), and preprocessed the image on the ENVI 5.3, including radiometric calibration and atmospheric correction of thermal infrared data and multispectral data to eliminate the errors from sensors and atmosphere. Then, we selected a suitable algorithm to retrieve the LST using the remote sensing image. At present, there are 4 methods: radiative transfer equation algorithm (RTE) (32), split window algorithm, single-channel algorithm, and multi-channel algorithm. Many previous studies utilized RTE and obtained accurate results (33–35). Thus, we determined the RTE method to retrieve the LST of the study area. According to the steps of the method, we converted the digital number of all the satellite image bands to at-satellite reflectance using the gray value of the raw data first. Then, we calculated the Normalized Difference Vegetation Index (NDVI) (36) and retrieved the brightness temperature of the blackbody using RTE. Finally, the LST map was obtained by the Planck formula (Figure 2A), and 5 zones showing the spatial distribution of heat and cooling islands in Figure 2B were classified using the mean-standard deviation method (37).

### Classification of Land Use

**Figure 3:**
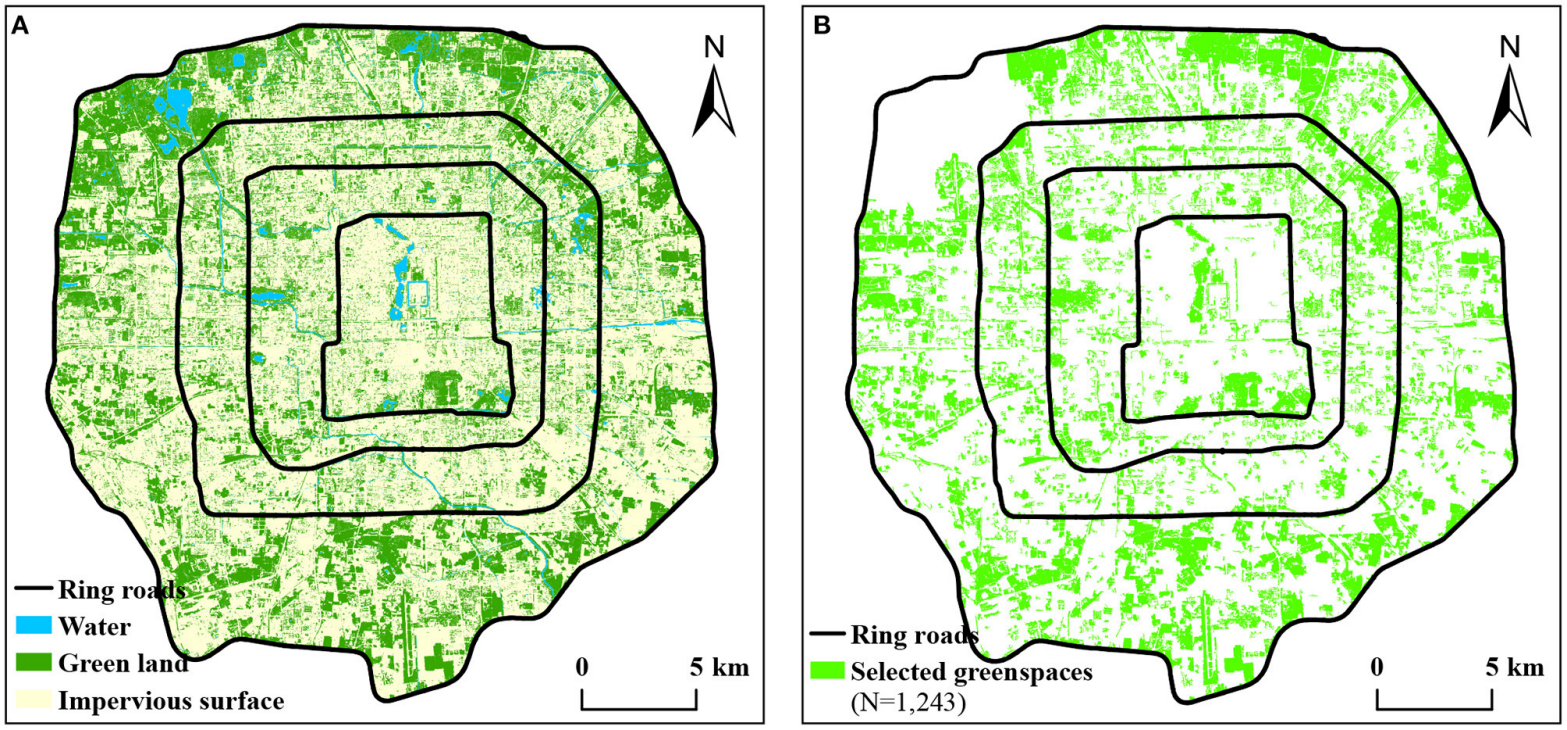
Spatial distribution of land use in the study area **(A)**, green spaces selected as the study objects **(B)**.

The land use data used in this paper are the global land cover in 2017 of 10 m accuracy published by the Tsinghua University (38). The acquisition time of the data is the same as that of remote sensing data used to retrieve LST. The data cover 10 categories of land use, and the study area involves 8 of them. Then, we combined farmland, forest, shrubland, and grassland into a new green land layer, combined water and wetland into a new water layer, and combined impervious surface and bare land into a new impervious surface layer (Figure 3A). After that, we merged the new green land layer and the new water layer within 30 m into the green space layer. Previous research indicated that the minimum area of the parks with a cooling effect in Beijing is around 1–3 hm^2^ (39). To eliminate interference, the green spaces with the area below 1 hm^2^ were deleted. In addition, the Summer Palace in the northwest of the study area is connected with Haidian Park, Liangshan Park, and some orchards, becoming a vast green space with an area of more than 1,900 hm^2^. The size of this green space is much larger than other green spaces. To make mathematical statistics more scientific, the green space was deleted. Finally, 1,243 green spaces were selected as the study objects (Figure 3B).

### Definition of Cooling Indicators and Landscape Features

**Figure 4:**
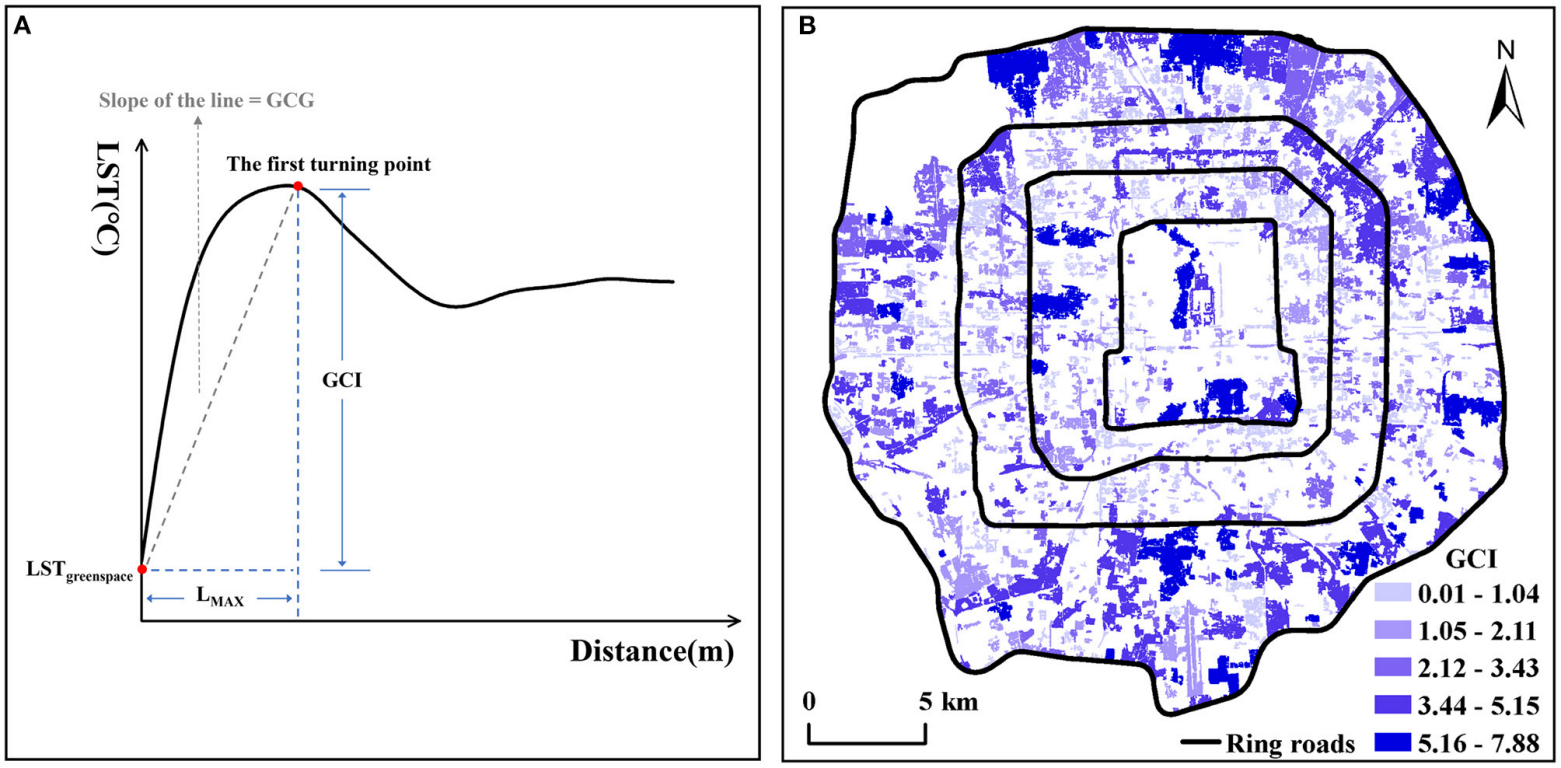
Average LST schematic diagram of green space and surrounding impervious surface **(A)**, the spatial distribution of 1,157 green spaces with different degrees of GCI **(B)**.

The cooling capacity of green space is evaluated by several indicators. Reflecting the amplitude of reducing LST of the surrounding impervious surfaces, green space cooling intensity (GCI) is one of the core indicators. We used the buffer method (25, 40) to define the GCI. We set 10 ring buffers with 60 m width for each green space from their boundary, and the average LST of impervious surface in each buffer was calculated. It is worth noting that we did not calculate the average LST of the entire buffers as in some previous studies to reduce the impact of other green spaces in the buffers on LST. Then, the curve was drawn in Figure 4A based on the data of a certain green space. The horizontal axis is the distance between the green space boundary and the buffer. And the vertical axis is the average LST of the green space and the impervious surface in a buffer. According to previous studies (41–43), we quantified the maximum distance of the green space cooling effect (L_MAX_) and the green space cooling intensity (GCI). L_MAX_ was confirmed as the distance from the first turning point of LST to the green space boundary; GCI was defined as the difference between the impervious surface temperature at L_MAX_ and the average LST of the green space. According to the definition, 1,157 green spaces' cooling intensity was calculated as shown in Figure 4B. Besides, we also defined and quantified other indicators reflecting green space cooling capacity or landscape features, such as the green space cooling area (GCA) (28), the green space cooling efficiency (GCE) (44), the green space cooling gradient (GCG) (28), and the green space shape index (GSI), as can be seen in Table 1.

**Table 1:** Definition of cooling and landscape indices.

**Cooling indicators and landscape features**	**Abbreviation**	**Definitions**	**Meaning**	**Unit**
The maximum distance of green space cooling effect	*L*_*MAX*_	*L*_*MAX*_ is the distance from the green space boundary to the buffer with the first turning point	Cooling range of the green space	*m*
Green space cooling intensity	*GCI*	*GCI* is the difference between the impervious surface temperature at *L*_*MAX*_ and the average *LST* of the green space $GCI=T_{LMAX}-T_{G}$ Where, *T*_*LMAX*_ is the impervious surface temperature (°C) at *L*_*MAX*_; *T*_*G*_ is the average *LST* of the green space	Amplitude of lowering temperature of surrounding impervious surface	°C
Green space cooling area	*GCA*	*GCA* is the buffer area with the maximum cooling distance from green space boundary	Cooling coverage	m^2^
Green space cooling efficiency	*GCE*	*GCE* is the ratio of the green space cooling area to green space area $GCE=\frac{GCA}{GA}$ Where, *GA* is the green space area (*m*^2^)	Area cooled by per unit area of green space	/
Green space cooling gradient	*GCG*	*GCG* is the ratio of green space cooling intensity to the maximum distance of green space cooling effect $GCG=\frac{GCI}{L_{MAX}}$	Temperature cooled per unit distance of green space cooling effect	°C/m^2^
Green space shape index	*GSI*	*GSI* is the ratio of perimeter to area of green space $GSI=\frac{C_{G}}{GA}$ Where, *C*_*G*_ is the perimeter (*m*) of green space	Shape complexity of green space	*m* ^−1^

### Identification of Green Space Clusters Based on Cooling Effect and Spatial Feature

We used the K-means clustering algorithm to identify different green space clusters with different cooling effects or spatial features. First, we normalized the 5 selected factors to ensure the accuracy of the subsequent calculations. Then, we found the appropriate K-value before analysis. Yuan and Yang indicated that compared with other methods of finding K-value, the Elbow method has a faster speed for less and uncomplicated data with the same accuracy (45). Therefore, this paper used python programming to obtain the K-value by the Elbow method. The result showed that when the K-value is 4, the sum of squared errors (SSE) is smaller. Finally, we took 4 as the suitable K-value and divided 1,157 green spaces into 4 clusters with different cooling effects and spatial features.

### Analysis of the Green Space Cooling Contribution to Surrounding Communities

To explore whether the cooling effect of green spaces is enjoyed by surrounding communities, we defined the concept of green space cooling contribution for community residents (GCC) for the first time. The GCC is the product of the community population per unit of green space cooling area (GCA) and green space cooling intensity (GCI). The community population, located in cooling coverage, is the product of the total number of households in communities and the average population per household. We used big data crawling software, Octoparse, to obtain the total number of households and detailed addresses of all communities in our study area from the Anjuke website (https://beijing.anjuke.com), which is the largest online real estate trading website in China. Then, through python programming, we utilized Baidu Geocoder API to convert the detailed addresses of communities into coordinates recognized by ArcGIS. Finally, the community points data with the total number of households and the accurate spatial location was obtained. It is worth noting that all spatial data, such as remote sensing images, land use data, the community points data, all used the WGS-1984 coordinate system to be calculated seamlessly together in ArcGIS.


(1) $$GCC=\frac{N_{h}\times N_{P}\times GCI}{GCA}$$

Where **N**_**h**_ is the total number of households located in cooling coverage; **N**_**P**_ is the average population per household. According to the *Beijing statistical yearbook 2018*, the **N**_**P**_ of the study area is 2.52 (46). And **GCI** is the green space cooling intensity; **GCA** is green space cooling area.

## Results

### General Characteristics of LST and Its Corresponding Land Use

Combined with the LST of the study area in Figure 2A and cold-hot islands distribution in Figure 2B, we identified many hot spots which are mainly distributed in the old town, industrial and logistics areas, such as the central and southern part of the 2nd Ring Road, the southeast part of the 3rd Ring Road to the 5th Ring Road, and the southwest part of the 4th Ring Road to the 5th Ring Road. The LST in these areas is 35–40°C, and in some regions, its LST is above 45°C, which showed that the health condition of the urban thermal environment was worrying. Moreover, we found that the LST in the north and northwest of the study area is significantly lower. The low LST was also found in the northeast from the 4th Ring Road to the 5th Ring Road. Some parks, golf courses, and nursery gardens are distributed in these areas. In addition, there are many cold spots scattered in the city, mainly some comprehensive parks, riverside parks, and gardens adjacent to large buildings or communities. These areas are cool and comfortable for residents in summer.

According to the statistics in ArcGIS, we found that the average LST of the study area is 33.14°C, while the maximum LST is 51.88°C, and the minimum LST is only 22.43°C. The standard deviation (std) is 3.30. And in terms of the proportion of cold-hot islands area, the average temperature region accounted for the largest proportion which is 39.28% and the heat islands area accounted for 30.89% which is also higher than that of cold islands (29.83%). In addition, from the perspective of land use, the average LST of green spaces is significantly lower than that of impervious surfaces. The average LST of pure green land (about 20,257.29 hm^2^) is 31.09°C; the average LST of water (about 915.21 hm^2^) is 27.83°C and the average LST of impervious surface (about 45,375.93 hm^2^) is 34.46°C. It suggested that the green space, accounting for only 30% of the study area, reduces the average LST of the whole area by 1.32°C. So, it can be seen that urban green spaces have a strong cooling effect and benefit the thermal environment health of the city.

### Correlation Between Spatial Features and Cooling Effect

Previous studies showed that the spatial features of green spaces were correlated with their cooling effect (27, 47, 48). To verify the conclusion, Spearman's correlation analysis between them was performed. We selected the area (GA), shape index (GSI), and perimeter (C_G_) to describe the spatial features of green spaces; the cooling area (GCA), cooling intensity (GCI), cooling gradient (GCG), and the maximum cooling distance (L_MAX_) to describe the cooling effect of green spaces. The results of the correlation analysis are shown in Table 2.

**Table 2:** Correlation analyses between cooling indicators and spatial features of green space.

	**GCI**	**GCA**	**GCG**	**L_**MAX**_**
	**(°C)**	**(hm^**2**^)**	**(**°**C/m)**	**(m)**
GA (hm^2^)	0.473^**^	0.875^**^	0.505^**^	0.367^**^
GSI (m^−1^)	−0.650^**^	−0.456^**^	−0.549^**^	−0.477^**^
C_G_ (m)	0.370^**^	0.820^**^	0.358^**^	0.214^**^

** ,*indicate significant at the 1 and 5% levels, respectively.

**Figure 5:**
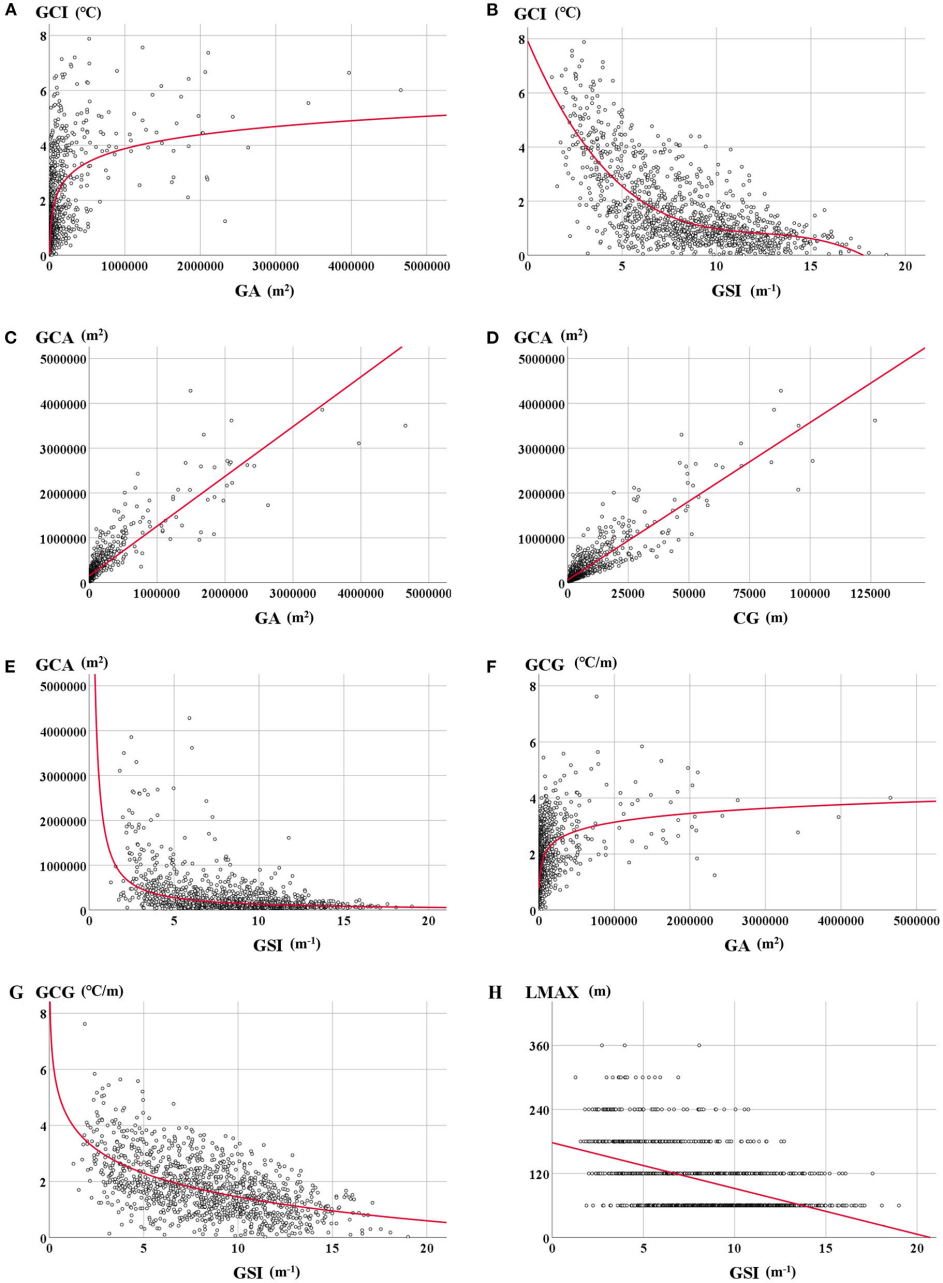
Relationship between dependent (GCI) and independent [GA: **(A)**, GSI: **(B)**], relationship between dependent (GCA) and independent [GA: **(C)**, C_G_: **(D)**, GSI: **(E)**], relationship between dependent (GCG) and independent [GA: **(F)**, GSI: **(G)**], relationship between dependent (L_MAX_) and independent [GSI: **(H)**]. The regression line using the ordinary least squares method is presented in red. The regression models were selected among linear, logarithmic, and power functions with the largest R^2^ value.

There is a significantly positive correlation (*p* < 0.01) between GA and GCI, indicating that the larger the green space area, the larger the cooling temperature, which is consistent with Li et al. (49). But the fitting curve in Figure 5A showed that with the increase of GA, the change rate of GCI gradually decreases. That is, after reaching a certain degree (about 50 hm^2^), if GA continues to increase, the change of GCI will be no longer obvious. Due to the scarcity of land in metropolitan areas, the size of green spaces is not recommended to be more than 50 hm^2^. C_G_ also exerts a positive effect on GCI (*p* < 0.01), but the relationship between them is weak (rho < 0.4), which indicated that the green space perimeter has little influence on the degree of cooling temperature. However, GSI is negatively correlated with GCI (*p* < 0.01), which showed that the more complex the green space shape, the smaller the cooling temperature. In Figure 5B, when the GSI value is larger than 17, the GCI reaches 0, and the green space cooling effect disappears.

GA and C_G_ are significantly in positive correlation with GCA (*p* < 0.01), which shows that the larger the green space size, the larger the cooling coverage in Figures 5C,D. GCA is also significantly negatively correlated with GSI (*p* < 0.01), suggesting the more complex the green space shape, the less the cooling coverage (Figure 5E).

GA is also positively correlated with GCG (*p* < 0.01), and GSI is negatively correlated with GCG (*p* < 0.01), indicating the larger the green space area and the more regular of green space shape, the larger the cooling gradient. That is, if green space is larger and regular in shape, it will be more likely to cool the surrounding temperature quickly and efficiently (Figures 5F,G). But there is no obvious positive correlation between C_G_ and GCG (*p* < 0.01, rho < 0.4), which suggests that green spaces with large or small perimeters have the same influence on their cooling gradient.

Only GSI has a significantly negative correlation with L_MAX_ (*p* < 0.01), which suggests that the more regular the green space shape, the longer the cooling distance (Figure 5H). GA and C_G_ have a positive influence on L_MAX_, but the relationship between them is weak (*p* < 0.01, rho < 0.4). So, the green spaces with larger or small areas and perimeters have the same influence on their cooling distance.

In sum, green space area (GA) and shape index (GSI) possess an obvious influence on green space cooling capacity, especially on cooling intensity (GCI), cooling area (GCA), and cooling gradient (GCG). Green space perimeters (C_G_) have a weak correlation with cooling capacity except for GCA. The maximum cooling distance (L_MAX_) is only significantly correlated with GSI. Thus, it can be seen that the green spaces with larger areas and more regular shapes probably possess a better cooling capacity.

### Green Space Clusters With Different Spatial Features and Cooling Effects

**Figure 6:**
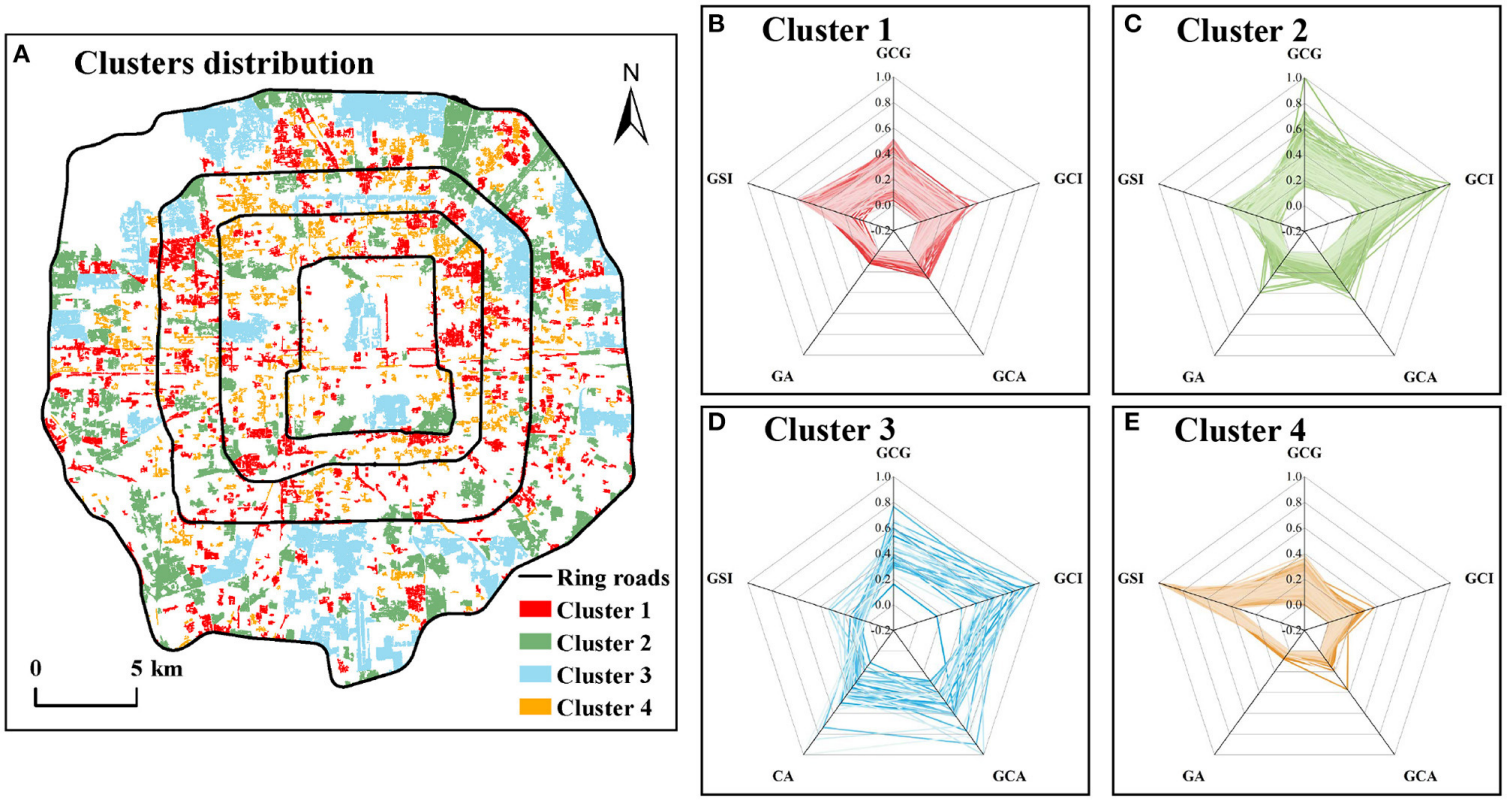
Spatial distribution of 4 clusters green spaces in different cooling effect **(A)**, Radar diagrams of landscape and cooling metrics for 4 clusters green spaces **(B–E)**.

GCI, GCA, and GCG, the core indicators of reflecting green space cooling capacity, together with GA and GSI, which have a higher correlation with the 3 core cooling indicators above, were selected as classification metrics. After the k-means method identification, 4 clusters (Figure 6A) were obtained from 1,157 green spaces and their features are shown in Table 3.

**Table 3:** Average characteristics of 4 green space clusters.

	**Green space quantity**	**GA (hm^**2**^)**	**GCA (hm^**2**^)**	**GSI (m^**−1**^)**	**GCI (**°**C)**	**GCG (**°**C/m)**	**L_**MAX**_ (m)**	**GCE**
Cluster 1	476	7.55	21.02	7.13	1.46	1.91	76.44	2.78
Cluster 2	189	26.70	59.62	4.47	4.07	2.90	140.34	2.23
Cluster 3	32	182.02	237.26	3.23	5.01	3.30	151.82	1.30
Cluster 4	460	3.76	14.66	11.56	0.70	1.10	63.64	3.90

Cluster 1 has the most green spaces, up to 476. These green spaces with an average area of 7.55 hm^2^ are mainly some large residential green spaces, greenbelts, and some small theme parks (Figure 6B). Their cooling indicators perform poorly: the average GCI is low, the cooling coverage is small, and the cooling distance is also close. But the GCE is higher. We believe that this cluster of green spaces is suitable for regions that possess some area for green space construction. And there is a comfortable thermal environment and low cooling demand.

Cluster 2 is mainly moderate-area parks, with an average area of 26.70 hm^2^ and smaller GSI (Figure 6C). The parks are evenly distributed in the study area. The GCI, GCA, L_MAX_, and GCG of these green spaces are higher, indicating that they can have an excellent cooling effect on the surrounding areas and create a more comfortable thermal environment for the metropolitan areas. But their GCE is relatively low. These green spaces need relatively adequate land and space, and they are recommended to be set in the areas with high UHI.

Cluster 3 is mainly large parks, with an average area of 182.02 hm^2^ (Figure 6D). They are primarily comprehensive or ecological parks distributed in the outer zone of the study area, and a small number of green spaces are historical gardens located in the inner and middle zones of our study area. These green spaces of the cluster have the largest GCI, GCG, GA, GCA, and L_MAX_, suggesting they, with the perfect cooling capacity, can reduce LST over long distances and can rapidly mitigate UHI of the surroundings. However, the GCE and the GSI are the smallest among all clusters, indicating most green spaces are more regular in shape, and the cooling efficiency is the lowest. In short, these green spaces have an excellent cooling effect, but the size is enormous, which is suitable for the region with more considerable cooling demand and sufficient area for green space construction.

Cluster 4 is mainly attached green space with the smallest area, including residential green space, green belts, green space attached to urban roads, and so on (Figure 6E). These green spaces are dominated by GSI and GCE, and other indicators perform poorly, such as GCA, GCI, GCG, and L_MAX_. They are the smallest among all clusters, suggesting their cooling capacity is extremely limited. Therefore, these green spaces are recommended to be located where needs little cooling demand.

### Green Space Cooling Contribution to Communities

#### Characteristics of Different Levels of Green Space Cooling Contribution

**Figure 7:**
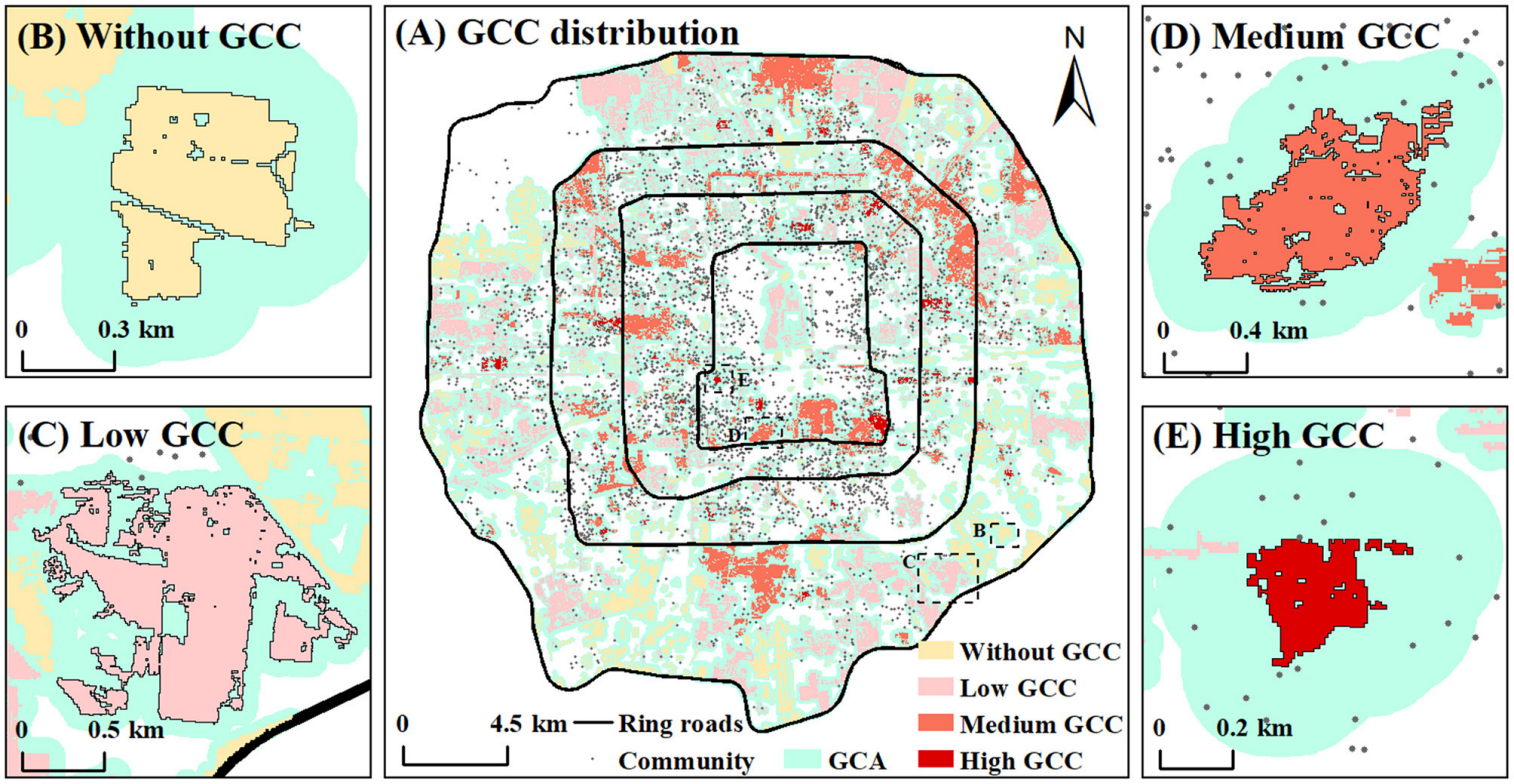
Spatial distribution of green spaces with different GCC levels **(A)**, examples of green spaces with different GCC levels **(B–E)**.

**Figure 8:**
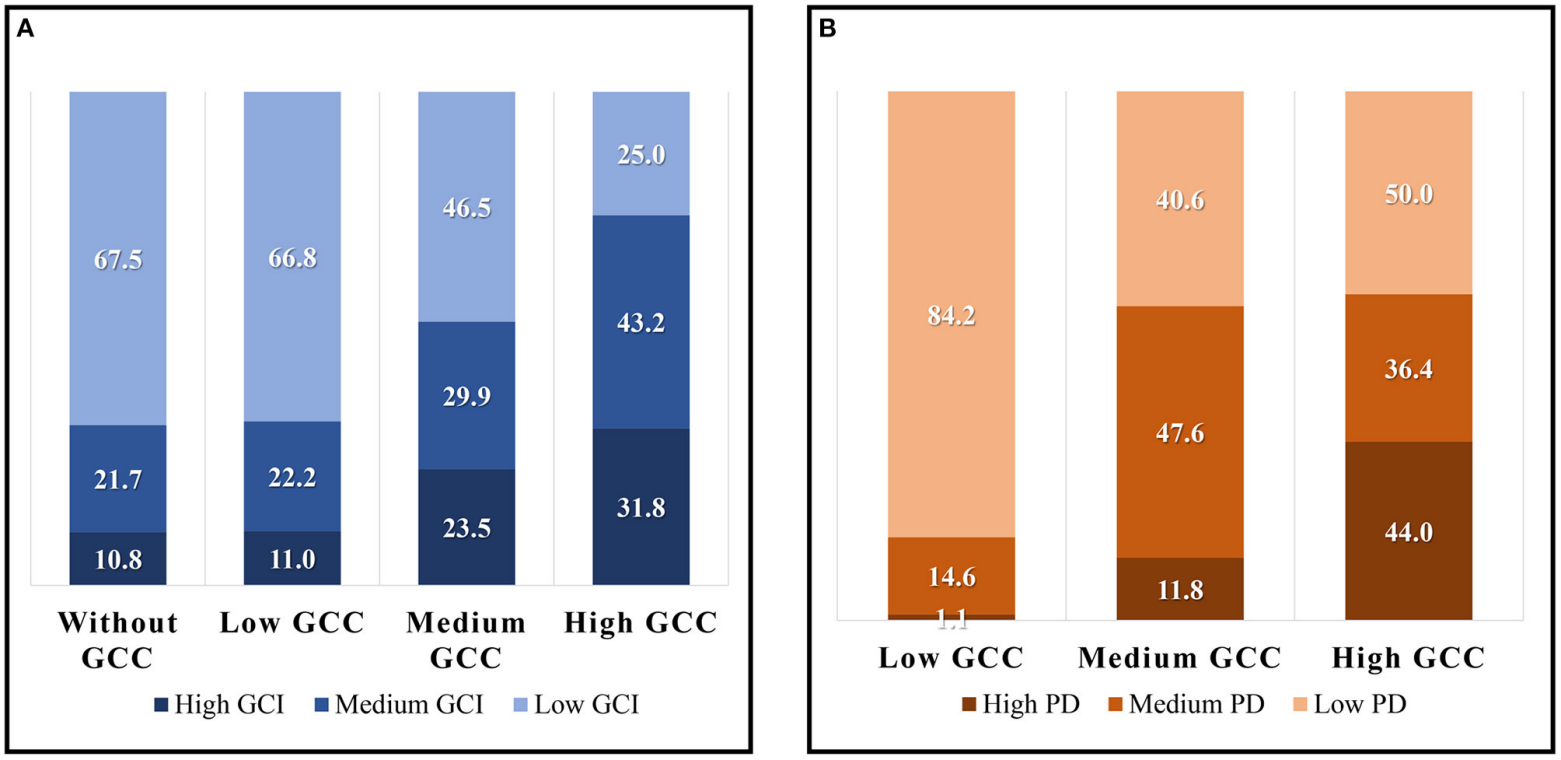
The proportion of green space with low, medium, and high GCI in different GCC groups **(A)**, the proportion of green space serving low, middle, and high population density in different GCC groups **(B)**.

According to the formula in formula 1, the cooling contribution for the community of 1,157 green spaces (GCC) was obtained, of which 489 green spaces have no community in their cooling coverage. That is, they do not have any GCC for communities. The rest of the green spaces were divided into three categories: low GCC, medium GCC, and high GCC according to the Natural Breaks (Jenks) method (Figures 7A, 8).

The 489 green spaces without contribution are mainly distributed in the east, southeast, and south of the outer zone in the study area. Their average area is small, only 8.25 hm^2^, which leads to their poor cooling capacity. The GCA and the L_MAX_ of the green spaces are the smallest among all categories, and their GCI is also smaller. For example. 67.5% of these green spaces possess low GCI in Figure 8A. Therefore, except no distribution of communities around the green spaces, the poor cooling effect and small cooling coverage might be the important reasons why they do not have any cooling contribution. Besides, there are 32.5% green spaces with middle or high GCI, suggesting that they possess a great cooling capacity and might become potential areas for new community construction. For example, Haitang Park (Figure 7B), located in the southeast of the outer zone, has an excellent cooling capacity and more extensive cooling coverage, but there was no community around it in 2017. According to the plan, several new communities will be distributed around it in the future.

There are 473 green spaces with low GCC, of which the larger ones are distributed in the outer zone, and the smaller ones are mainly distributed in the inner zone and middle zone. The average area of these green spaces is large, but their cooling capacity performs poorly. The GCI and L_MAX_ of most green spaces are very low, and they also serve smaller communities. So the low cooling contribution of these green spaces might be due to the lower amplitude of reducing LST as well as the small population of the surrounding communities. Significantly, 15.7% of the green spaces are surrounded by large communities and residents, but their cooling capacity is very weak. The cooling effect of these green spaces is the key that needs to be improved in the future. While 33.2% of the green spaces with middle or high GCI, such as Hongbo Park in Figure 7C, have only a few residents around them. They might play an important role in cooling the temperature for new communities in the future.

There are 187 green spaces with a moderate cooling contribution to the surrounding communities. And they are mainly distributed in the middle zone. The average area and GCA of these green spaces are the largest among all categories, and GCI and L_MAX_ are large too, which indicates that most of these green spaces have a perfect cooling performance. At the same time, a lot of communities are distributed around them, such as Taoranting Park in Figure 7D, thus, they have a good performance on cooling contribution to the surrounding communities. But there is still 46.5% of these green spaces needing to improve their cooling capacity to serve the surrounding communities better.

Only 44 green spaces have a high cooling contribution to the surrounding communities, and their spatial distribution is relatively scattered. Xuanwu Art Park is a typical green space with high GCC (Figure 7E). Although the area of these green spaces is the smallest, only 8.02 hm^2^, they have the perfect cooling performance. The GCI, GCE, and L_MAX_ of 75% of these green spaces perform perfectly among all green spaces. However, 11 green spaces surrounded by a large number of residents have a weak cooling effect, which should be improved in the future. Meanwhile, there are 6 green spaces with the highest cooling capacity with a few residents around, so they can become the potential areas to provide cooling services for new communities in the future.

#### Characteristics of Green Space Cooling Contribution in Different Urban Areas

**Figure 9:**
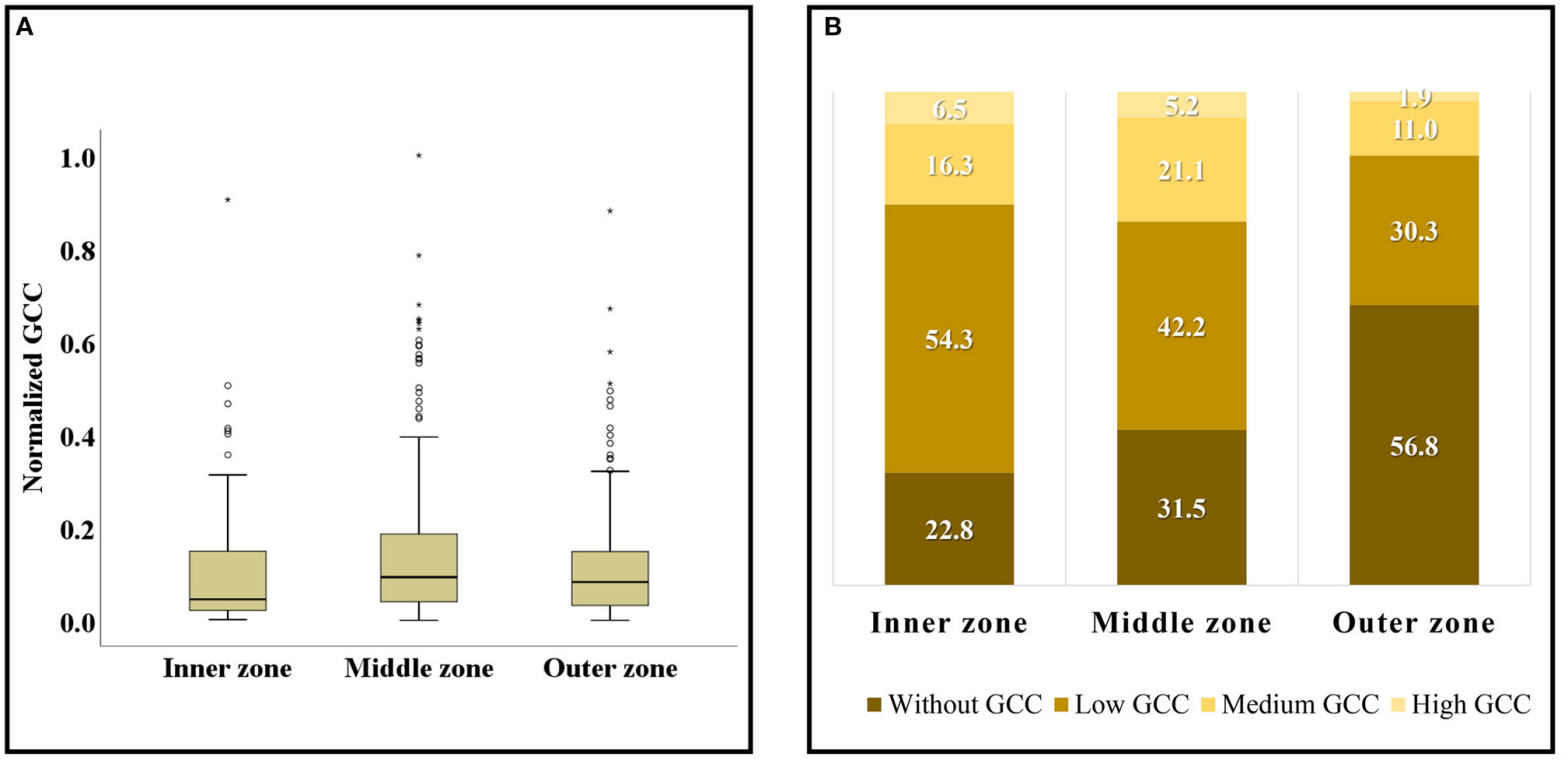
The box diagram of GCC distribution in different urban zones **(A)**, proportion of green spaces with different levels of GCC in different zones **(B)**. Hollow circles ° represent mild outliers; asterisks * represent extreme outliers.

Generally, there are 668 green spaces with GCC (Figure 9A). The GCC performance of the green spaces in the middle zone is the best, slightly better than that in the outer zone, and the worst in the inner zone. It is worth noting that the median value of the inner zone is lower than the bottom value of the middle zone and outer zone, indicating that the GCC of most green spaces in the inner zone performs very poorly. In terms of dispersion, the middle zone is also the largest, and the inner zone is slightly larger than the outer zone.

From the proportion of various levels of GCC in each zone (Figure 9B), the sum of levels of low GCC and no GCC accounts for more than 70% in all zones of which the value of the outer zone is the highest up to 87.1%. In addition, the quantity of green spaces with high GCC and low GCC accounts for the most in the inner zone; the quantity of green spaces with medium GCC accounts for the most in the middle zone; while the quantity of green spaces with no GCC accounts for the most in the outer zone up to 56.8%. It is concluded that most green spaces in the inner zone are with GCC, but the most value is lower. About 70% of the green spaces in the middle zone are with GCC, and the proportion of the medium and high levels of GCC is the largest, so the GCC performance in the middle zone is the best. However, due to more than half of the green spaces in the outer zone are not with GCC and the proportion of medium and high GCC is the least, the GCC performance of the outer zone is the worst.

#### Characteristics of Different Clusters' Green Space Cooling Contribution

**Figure 10:**
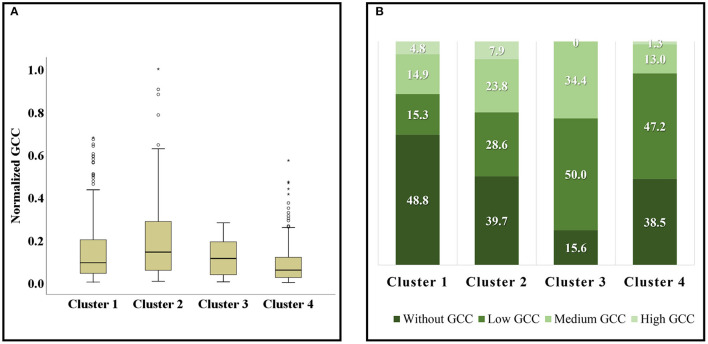
The box diagram of GCC distribution in different clusters **(A)**, the proportion of green spaces with different levels of GCC in different clusters **(B)**. Hollow circles ° represent mild outliers; asterisks * represent extreme outliers.

Among the 668 green spaces with GCC (Figure 10A), the GCC of cluster 2 is the highest, while the GCC of cluster 3 is slightly smaller than that of cluster 1, and the GCC of cluster 4 is the worst. Specifically, the reason the GCC of cluster 4 is worse than that of cluster 1 might be a small size, poor cooling intensity, and small cooling area of green spaces in cluster 4. Therefore the cooling capacity is so small not to mitigate the heat island effect of surrounding communities. In addition, although the size and cooling capacity of cluster 3 are larger than that of cluster 2, most of the green spaces of cluster 3 are distributed in the outer zone and serve a small number of communities and populations, so its GCC performance is not as good as that of cluster 2.

From the distribution of different levels of GCC in each cluster, nearly half of the green spaces in cluster 1 are not with GCC, and the value also reaches about 40% in cluster 2 and cluster 4 (Figure 10B). Only cluster 3 is relatively low—just 15.6%. Moreover, more green spaces in cluster 2 and cluster 3 are with medium- and high-level GCC. But cluster 4 has the least green spaces with medium and high GCC. In short, cluster 3 performed best in the cooling contribution for communities, followed by cluster 2, cluster 1, and cluster 4. It can be seen that the green space cooling contribution varies with spatial feature and cooling capacity.

## Discussion

### The Cooling Effect of Green Space in the Metropolitan Area

This paper found that urban green space can produce a cooling effect and significantly mitigate UHI, which has been confirmed by many studies (50, 51). However, it is worth noting that the cooling effect of green space is related to the distance from the green space boundary. That is, with the increase of the distance, the change of the effect becomes lower and lower. When the turning point of distance appears, the cooling effect changes to zero. It showed that the cooling range of green space is not infinite, which is consistent with other research results (25). Besides, we also found that GA and GSI were significantly correlated with GCI, which is consistent with Qiu et al. (39) and Shah et al. (44). But some studies believed that there was no apparent relationship between them. The number of samples analyzed in these studies is only several dozens (28, 48). The lack of adequate samples may affect the accuracy of the studies and lead to the inconsistency of these results. These studies also pointed out that green spaces with small size, a high proportion of water area, and high NDVI usually possess higher GCI than those large green spaces with only forest and grass (34, 43, 50). Our research also confirms the view at the case level. The GCI of the Xuanwu Art Park (7.8 hm^2^) with water accounting for 20% of the area and dense vegetation is much higher than that of some large-sized nurseries (more than 100 hm^2^) in the southwest and southeast of the study area, where the vegetation is sparse and growing.

This paper also confirmed that GA and C_G_ are positively correlated with GCA, which was consistent with Lin et al. (52). The larger the area and perimeter, the larger the contact area between the green space and the surrounding impervious surface, boosting the heat exchange, thus, increasing the cooling coverage. After that, GA and GSI are also significantly correlated with GCG, which is different from Chen et al. (28) and Qiu et al. (53). They believed that GCG is related more to the water area. In conclusion, we believe that green space planning and rebuilding should take the specific cooling needs of the region into consideration, such as cooling coverage, cooling intensity, and cooling gradient. Based on the specific cooling needs, the spatial features and construction elements of green space should be scientifically arranged to effectively alleviate the UHI and create a comfortable and healthy thermal environment in summer.

### Rational Spatial Arrangement and Optimization of 4 Green Space Clusters

We found that the green space with a larger area and more regular shape possesses higher GCI, GCA, GCG, and L_MAX_ except for GCE. However, due to the scarcity of land in large cities, especially in metropolitan areas, it is necessary to weigh the relationship between the size of green space and the cost of green space planning and construction (54, 55). With the premise of enjoying good green cooling services, we would like to save funds and costs as much as possible. Therefore, it is necessary to fully understand and make the most of the cooling advantages and features of different green space clusters to adjust and optimize the spatial distribution of green space to realize the optimal green space cooling effect. For example, in densely populated urban fringe areas with large cooling demands and sufficient construction space, it is recommended to build large ecological parks, like the green spaces in cluster 3. These green spaces have the best cooling capacity to mitigate UHI effectively, and also possess important ecosystem service functions. Moreover, in large commercial office areas, communities, and industrial areas with high cooling demands and limited available land, some medium-sized urban parks can be arranged, like the green spaces in cluster 2. These green spaces can be designed with a certain area of water and dense forest vegetation, which not only have a better cooling effect and benefit the health of the surrounding thermal environment to a great extent but also provide entertainment benefiting citizens' mental health. After that, for some communities and office areas lacking available land and large green spaces, some fragmented green spaces in these areas can be integrated into a blue-green infrastructure with several hectares and relatively regular shape, which can also give a good cooling effect, like the green spaces in cluster 1. In sum, the cooling demand of citizens and the cooling supply of different green space clusters should be considered and matched in urban green space planning and construction (56, 57).

### The Different Performance of Green Space Cooling Contribution to Surrounding Communities

From the perspective of different levels of GCC, we found that many green spaces were with low GCC or without GCC, but the surrounding of some of these green spaces was the potential location for new communities in the future. Meanwhile, despite the high value in GCC, some green spaces still need to improve their cooling capacity to meet the cooling requirement. Thus, in the process of green space planning and repairing, GCC should be improved according to the green space conditions, cooling demand from residents, and the reasons for this phenomenon.

According to the above results, more than 80% of the green spaces were without GCC or with low GCC. We believe that there are mainly two reasons for this phenomenon. First, there might be no community distribution within a certain distance around the green spaces or the size of the community population is small, that is, the green space cooling effect served too few residents. Second, there are communities around, but the performance of the cooling capacity of these green spaces is very poor. To be specific, these green spaces' cooling range cannot cover enough surrounding communities and their cooling intensity is also weak. Based on the two reasons, we proposed the following strategies to improve GCC. For the first reason, we recommended that according to urban planning, the surrounding of the green spaces with excellent cooling effects can be used as a potential site for new communities. For the second reason, we advised that the green spaces should improve their cooling capacity by modifying their spatial or landscape features to promote their cooling contribution. According to the above results and other studies, it can be enhanced effectively *via* increasing the green space area (GA), water area (58), living vegetation volume (LVV) (59), and the area proportion of dense trees and shrubs (60), and reducing boundary complexity (GSI). Moreover, as the above illustrated, there were also some green spaces with middle and high GCC that need to be worthy of attentions. They are surrounded by a high-density population and perform poorly in cooling capacity. We recommended these green spaces should refer to the promotion methods of the second reason to advance their GCC by increasing their cooling effect. Finally, we suggested that the level of GCC should be taken into consideration in future green space planning and construction, and GCC can be taken as one of the evaluation indicators of whether the green space should be repaired and whether the surrounding of the green space can be suitable to build new communities.

From the perspective of different city zones, we found that there is a variance in green space cooling contribution among these regions. That is, the performance of GCC in the middle zone is the best and the performance of the inner zone is slightly better than that of the outer zone. Previous studies also confirmed that real regional differences have existed in the green space cooling effect (14), which indicated potential unfairness in green space cooling services among city zones. We guessed the reasons for this phenomenon might be related to the history of urban development. As an old town, the inner zone was saturated before 2000. The green space construction at that time did not receive enough attention. Many green spaces with small or medium size and worse cooling capacity are surrounded by communities, which leads to many green spaces with low GCC appearing in the inner zone. However, the middle zone is the key construction area from 2000 to 2015. With the rapid increase of population in Beijing and the improvement of people's demand for urban green spaces, the government began to pay attention to the scientific construction of urban green spaces. Therefore, there are many modern parks and residential green spaces with larger sizes and good cooling capacity, which makes them perform better in cooling contribution. The outer zone is the key area of current urban construction. Although some green spaces have a large size and perfect cooling effect, the population density in this area is relatively low. Meanwhile, there are still many regions remaining to be developed and exploited. Thus, many green spaces are not with GCC.

Therefore, we suggest that the government and managers attach enough importance to the invisible and potential imbalance and unfairness and bridge it (61). The inner zone can make full use of the current urban renewal policy, and integrate the small green spaces around the community to form urban green infrastructure on a larger scale to produce a cooling effect fully. As for the green spaces in the middle zone, we should further improve the green space cooling contribution purposefully according to the evaluation results of the cooling effect. As for the outer zone, the results of GCC can be used as an essential basis for selecting the new community locations to improve the resident density around the green space with an excellent cooling effect.

### Research Limitations and Future Directions

Our research still exerts some limitations in the following aspects. First, the LST retrieved from the remote sensing data is the surface temperature at a specific static moment. In the future, if we obtain dynamic remote sensing data (or LST data) and population distribution data, we will get the GCC of the whole day, and have a deeper understanding of the green space cooling capacity and cooling services. Second, due to the limitation of fine resident distribution data, we replaced it with point data of the community from the real estate trading website. In the future, we will try to obtain more accurate population distribution data or vector data of communities and buildings including population information to make our research more scientific. Finally, the study only focused on the green space cooling service to the community. In the future, all regions with high-density populations in our study area also can be selected for research.

## Conclusion

So far, many studies have discussed the influencing factors of the cooling effect of green space (15, 29, 62). But few studies have discussed whether the cooling effect of green spaces is enjoyed by residents. Therefore, from a humanistic perspective, this study furthers our understanding of how the different locations in urban zones and different spatial features of green spaces affect their cooling effect and cooling contribution to the surrounding communities, which is crucial to improving the cooling capacity of green spaces in summer and creating a comfortable urban thermal environment.

Thus, we first investigated the overall characteristics and spatial distribution of LST in the study area by using the radiative transfer equation method. The results showed that the old town and industrial and logistics areas possessed the highest LST, while the parks and water possessed the lowest. And the area of the urban heat island is larger than that of the cooling island and the LST of water among all types of land use is the lowest (27.83°C). Accounting for only about 30% area of the study area, green spaces reduced the LST of the whole study area by 1.32°C. Then, we explored the correlation between the spatial features of green spaces and their cooling capacity. The results showed that the GA and GSI are significantly correlated with their cooling capacity, indicating that the green spaces with large areas and regular shapes generally have a good cooling effect. However, there is a threshold for the GA, which is about 50 hm^2^. Next, according to the spatial characteristics and cooling effect, 1,157 green spaces with cooling capacity are divided into 4 clusters. And the cooling supply of the 4 clusters was also discussed, respectively. Finally, we defined and mapped the green space cooling contribution to the surrounding community, and discussed the methods of green spaces with different levels of GCC to improve their contribution. It was found that 32.5% of green spaces without cooling contribution, 33.2% of green spaces with low contribution, and 23.5% of green spaces with medium contribution have good cooling capacity, but the surrounding population density is low, which is the potential area set for the new communities in the future; 25.7% of green spaces with low contribution and 11.8% of green spaces with medium contribution served a large population. But these green spaces' cooling capacity is poor, so it is vital to improve their cooling effect. Lastly, based on the urban spatial pattern and the distribution of green space cooling contribution, we further discussed the performance characteristics of GCC in different urban zones and different green space clusters. The study found that the GCC in the middle of the Beijing metropolitan area is significantly better than that of the inner and the outer zones. The green space clusters with moderate scale and high cooling effect have a higher GCC. In the future, the community layout could take green space cooling contribution into consideration to make full use of the optimal efficiency of the green space cooling effect on the health of the community thermal environment.

The study helps guide the green space planning in metropolitan areas to a certain extent and allows the government and planners to deeply understand the actual situation of the cooling effect of green spaces and their cooling contribution to the communities. In addition, it has certain practical significance for improving the ecosystem service of urban blue-green infrastructures and community health and wellbeing.

## Data Availability Statement

The datasets presented in this study can be found in online repositories. The names of the repository/repositories and accession number(s) can be found at: https://www.gscloud.cn/home; http://data.ess.tsinghua.edu.cn/fromglc2017v1.html; https://beijing.anjuke.com/community/?from=navigation.

## Author Contributions

HZ put forward the ideas, conducted experiments, wrote and modified the manuscript, and drew all figures and tables. WL perfected the initial ideas, provided guidance and suggestions, and did some checking. JZ provided guidance and suggestions. SS and XX solved some technical problems and provided suggestions. TH polished the writing of the manuscript. All authors contributed to the article and approved the submitted version.

## Funding

This study was supported by the MOE (Ministry of Education in China) Project of Humanities and Social Sciences (Grant No. 18YJC850016) and the Fundamental Research Funds for the Central Universities (2019ZY43).

## Conflict of Interest

The authors declare that the research was conducted in the absence of any commercial or financial relationships that could be construed as a potential conflict of interest.

## Publisher's Note

All claims expressed in this article are solely those of the authors and do not necessarily represent those of their affiliated organizations, or those of the publisher, the editors and the reviewers. Any product that may be evaluated in this article, or claim that may be made by its manufacturer, is not guaranteed or endorsed by the publisher.
